# 1-D nanoporous anodic alumina rugate filters by means of small current variations for real-time sensing applications

**DOI:** 10.1186/1556-276X-9-315

**Published:** 2014-06-25

**Authors:** Gerard Macias, Josep Ferré-Borrull, Josep Pallarès, Lluís F Marsal

**Affiliations:** 1 Department of Electronic, Electric and Automatics Engineering, Universitat Rovira i Virgili, Avda, Països Catalans 26, Tarragona 43007, Spain

**Keywords:** Nanoporous anodic alumina, Rugate filter, Photonic crystal, Fabrication, Nanostructuring, Current control, Sensing

## Abstract

A rugate filter based on nanoporous anodic alumina was fabricated using an innovative sinusoidal current profile with small current variation. The resulting structure consisted of highly parallel pores with modulations of the pore diameter along the pore axis and with no branching. The effect of the period time and the pore widening post-treatment was studied. From reflectance measurements, it was seen that the position of the reflection band can be tuned by adjusting the period time and the width by pore-widening post-treatments. We tested one of the rugate filters by infiltrating the structure with EtOH and water in order to evaluate its sensing capabilities. This method allows the fabrication of complex in-depth modulated nanoporous anodic alumina structures that open up the possibility of new kinds of alumina-based optical sensing devices.

## Background

Rugate filters are one-dimensional photonic crystals based on a smooth variation of the refractive index along the depth of the structure which results in a photonic bandgap (PBG) [1]. Unlike distributed Bragg reflectors (DBR), rugate filters display a single reflectivity band without harmonics or sidelobes. Thanks to this feature, rugate filters with complex optical response and multiple PBG can be fabricated by superimposing multiple refractive index profiles [1-3]. However, these filters are difficult to fabricate because the smooth variation of the refractive index is challenging and requires complex equipment. An interesting method for fabricating rugate filters is by means of electrochemically etched materials such as porous silicon (pSi). In porous materials, the refractive index depends on the porosity of the layer. Thus, pSi rugate filters have been fabricated thanks to the ease of porosity modulation by adjusting the electrochemical etching conditions [4-6]. Thanks to the porous nature of the resulting pSi rugate filters, these optical devices have been exploited for the development of highly sensitive detectors [7-12].

Another interesting material for the development of highly sensitive optical sensors is nanoporous anodic alumina (NAA) [13-21]. NAA is a nanostructured material obtained from the electrochemical etching of high-purity aluminum foils that has attracted much interest in recent years thanks to its unique structural properties. NAA consists of highly uniform and parallel pores with no branching. The interpore distance can be easily tuned by adjusting the voltage applied during the electrochemical etching, and the pore diameter can be adjusted by wet chemical etching in phosphoric acid [22]. Moreover, honeycomb structures of self-ordered pores can be obtained by the two-step anodization procedure [23]. However, porosity modulation with NAA has been challenging.

One of the first techniques used for pore modulation during the anodization was pulse anodization [24-26]. This technique consisted in combining mild and hard anodization regimes by means of step voltage variations. This allowed great changes in the pore diameter along the pore axis, but despite the fact that no optical characterization was performed, the combination of mild and hard anodization regimes would result in abrupt refractive index variations which are incompatible with the development of rugate filters. Another technique is cyclic anodization. This method was used to fabricate DBRs by applying a periodic voltage which resulted in well-defined layers with branched pores [27-29]. Lately, NAA photonic crystals fabricated with current control techniques have been reported [30,31]. However, these structures also showed branched pores.

In this work, we report a current control technique for the fabrication of NAA rugate filters. We have characterized the resulting structure and analyzed its optical response as a function of porosity by applying subsequent pore-widening processes. Finally, we tested the sensing capabilities of the NAA rugate filters by real-time monitoring the shift of the central wavelength in ethanol and deionized water.

## Methods

### Materials

Aluminum (Al) foil (thickness = 250 μm, purity = 99.999%) was purchased from Goodfellow (Huntingdon, UK). Oxalic acid (H_2_C_2_O_4_), ethanol (C_2_H_5_OH), acetone ((CH_3_)_2_CO), perchloric acid (HClO_4_), hydrochloric acid (HCl), and copper chloride (CuCl) were purchased from Sigma-Aldrich (Madrid, Spain). Double deionized (DI) water (18.6 MΩ, Purelab Option-Q, Elga, Marlow, UK) was used for all the solutions unless otherwise specified.

### Fabrication

**Figure 1:**
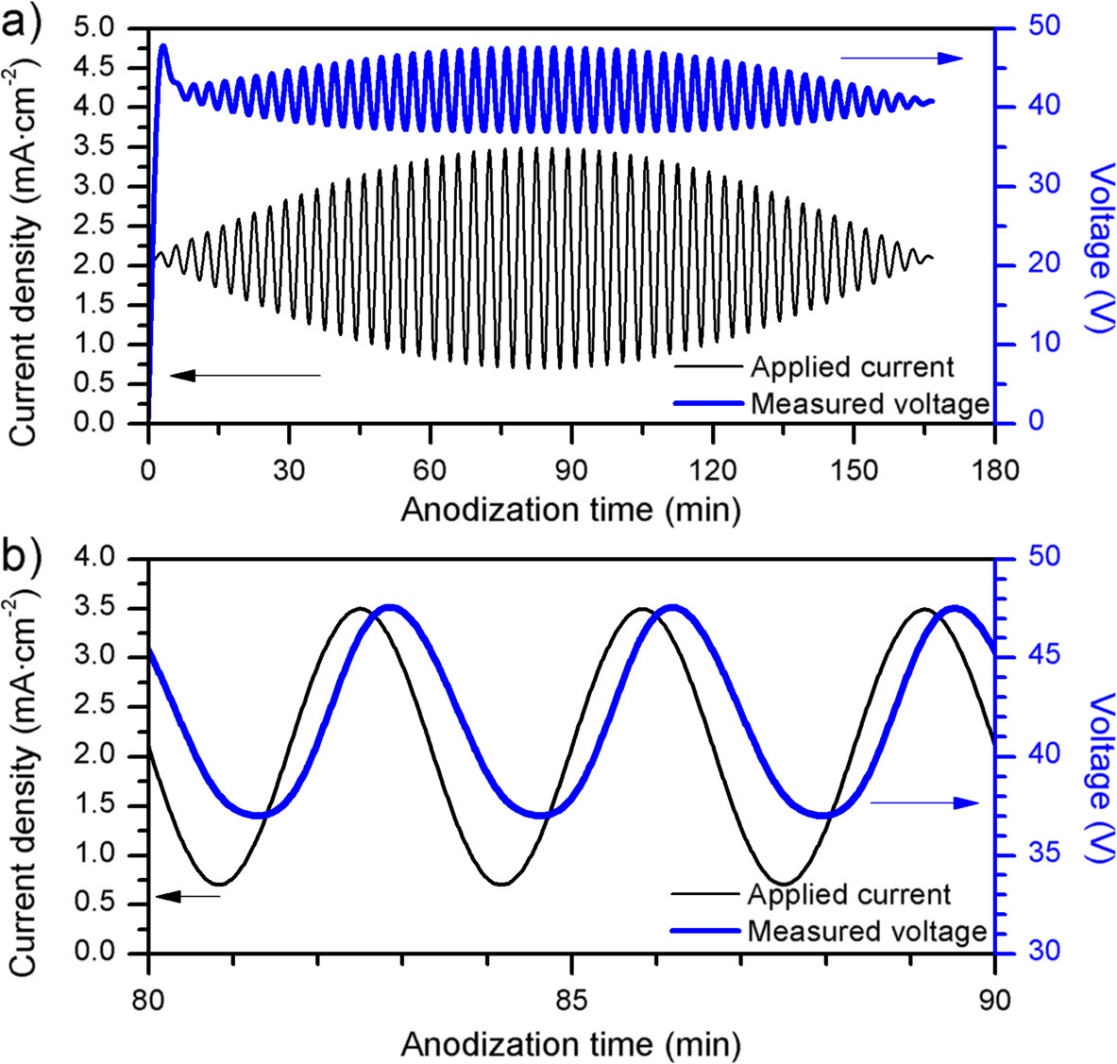
**Characteristic current and voltage evolution during the fabrication of an apodized NAA rugate filter. (a)** Full experiment and **(b)** magnification of the region with maximum amplitude of current profile.

Al substrates were first degreased in acetone and further cleaned with ethanol (EtOH) and DI water and dried under a stream of air. Prior to anodization, Al substrates were electropolished in a mixture of EtOH and perchloric acid (HClO_4_) 4:1 (*v*/*v*) at 20 V and 5°C for 4 min. During the electropolishing procedure, the stirring direction was alternated every 60 s. Then, the electropolished Al substrates were cleaned in EtOH and DI water and dried under a stream of air. Subsequently, the anodization of the aluminum in H_2_C_2_O_4_ 0.3 M at 5°C was carried out by applying an apodized current profile consisting of a DC component of 2.05 mA cm^−2^ with a superimposed alternating current (AC) sinusoidal component with variable amplitude. The amplitude of this AC component was modulated with a half-wave sinus profile with 1.45 mA cm^−2^ of maximum amplitude (see Figure 1a). We investigated the influence of the period (*T*) of the sinusoidal component on the optical characteristics of the obtained structures. Afterwards, different pore-widening post-treatments in H_3_PO_4_ 5% wt. at 35°C were performed for *t*_pw_ = 0, 5, 10, and 15 min in order to study the effect of porosity on the characteristics of the reflectance bands of the NAA rugate filters. Finally, Al bulk was selectively dissolved using a HCl/CuCl-saturated solution.

### Characterization

Scanning electron microscope (SEM) micrographs used for structural characterization of the NAA rugate filters were taken on SEM FEI Quanta 600 (FEI, Hillsboro, OR, USA). The optical characterization of the rugate filters was performed on a PerkinElmer UV/vis/NIR Lambda 950 spectrophotometer (PerkinElmer, Waltham, MA, USA). For the reflectance measurements, the spectrophotometer was coupled with the universal reflectance accessory (URA).

### Sensing experiment

Real-time measurements for the sensing experiments were performed in a custom-made flow cell. Reflectance spectra of the NAA rugate filter were obtained using a halogen light source and a CCD spectrometer (Avantes, Apeldoorn, The Netherlands). Light was directed to the surface at a normal angle through a fiber optic cable consisting of six illuminating waveguides and one reading waveguide coupled to an optical lens which focused the light on top of the NAA rugate filter. The light reflected by the rugate filter sample was collected by the reading waveguide and directed to the CCD spectrometer, which recorded a spectrum every 10 s.

## Results and discussion

### Structural characterization

**Figure 2:**
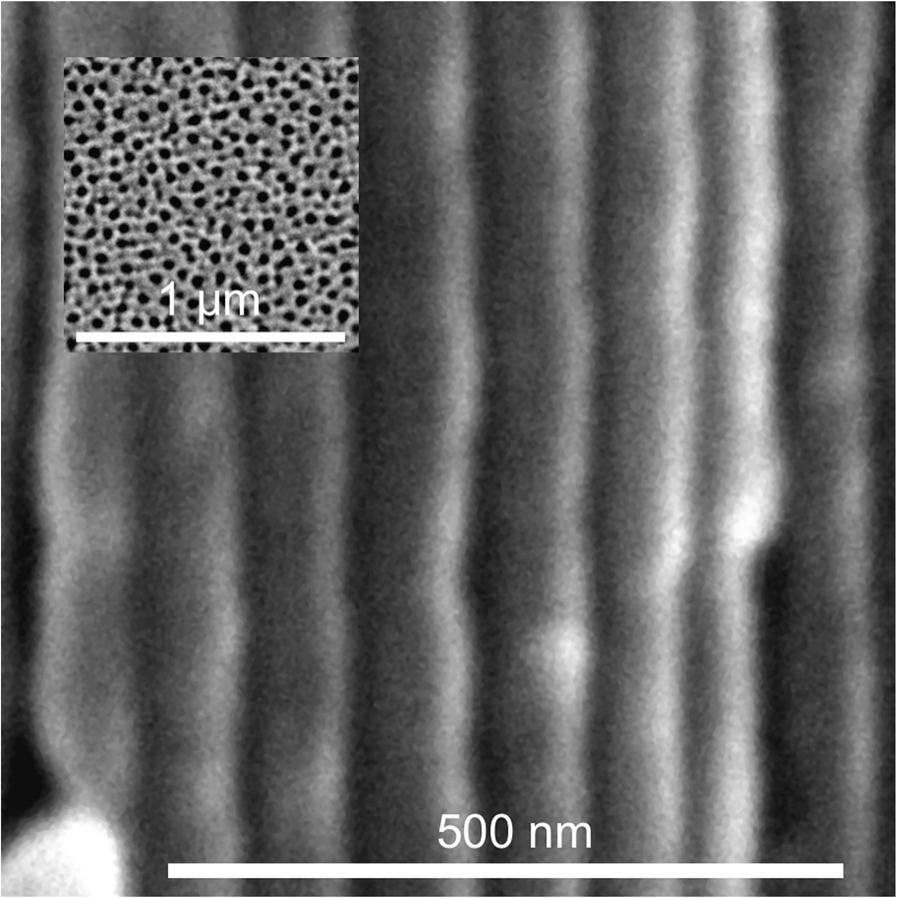
**Structural characterization.** Cross section SEM micrograph of a NAA rugate filter anodized for 300 cycles with an apodized sinusoidal current profile with a period of *T* = 200 s and a pore-widening post-treatment of *t*_pw_ = 15 min. Inset shows the top view of the structure.

Figure 1a shows the characteristic current and voltage evolution with time during the fabrication of NAA rugate filters. In this approach, we performed an apodization of the current profile in order to minimize the sidelobes in the reflectance spectra. Figure 1b shows a magnification of the area with the maximum current amplitude. We observed how the current density profile used throughout the experiments resulted in an initial transitory voltage (Figure 1a), which corresponds to the growth of the NAA barrier layer at the bottom of the pores, followed by an apodized sinusoidal voltage profile oscillating between 37 and 48 V with an average value of 41 V that resembles the applied current profile. A closer look at the electrochemical fabrication curves reveals a delay of the voltage with respect to the current. The resulting nanostructure is shown in Figure 2. The results presented here are for disordered porous alumina (Figure 2). Nevertheless, the narrow voltage range measured during our experiments would allow the fabrication of self-ordered rugate filters. The analysis of the cross-sectional micrograph of the NAA rugate filter reveals pore modulation without branching along the pore axis. This is due to the varying current profile (Figure 2) which produced a porosity gradient and, thus, a varying refractive index in depth.

### Central wavelength calibration

**Figure 3:**
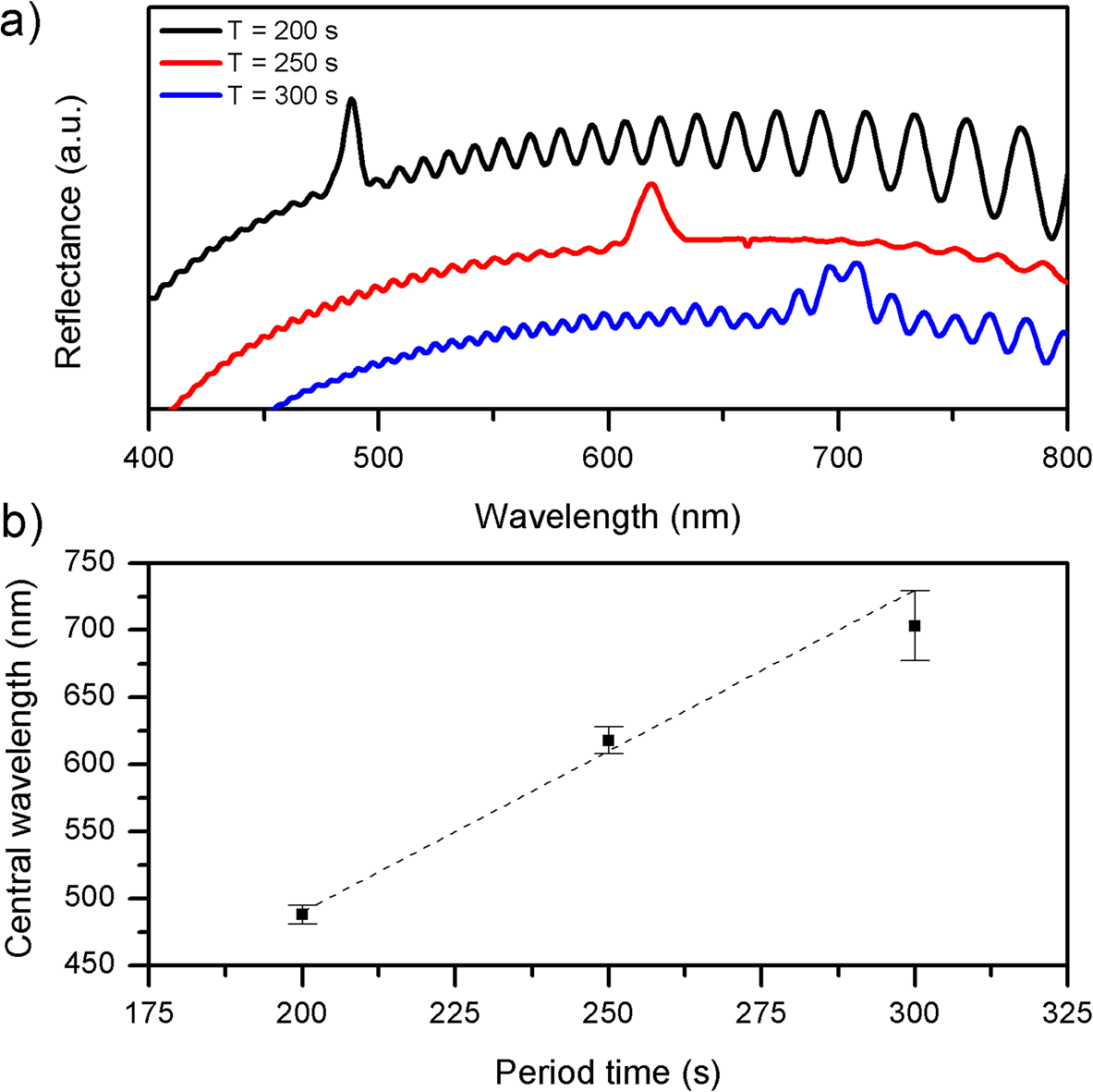
**Central wavelength calibration of NAA rugate filters. (a)** Reflectance spectra of NAA rugate filters anodized with a period of *T* = 200, 250, and 300 s for 50 cycles and **(b)** central wavelength position of the resonance band as a function of period time. The squares represent the central position of the resonance band, and the error bars correspond to the bandwidth.

In order to calibrate the position of the reflectance band, we fabricated three sets of samples with periods of *T* = 200, 250, and 300 s (Figure 3a). By increasing the period time, we increased the period of the pore diameter variations and, thus, tuned the position of the reflectance band. Another option would be to shift the current to higher values. However, we discarded this solution because of the higher potentials achieved which were beyond the self-ordering regime. As depicted in Figure 3b, shifting the period time allows linear tuning of the reflectance band at a rate of 2.4 nm s^−1^. Furthermore, the spectra show how longer periods result in wider bands.

### Effect of porosity

**Figure 4:**
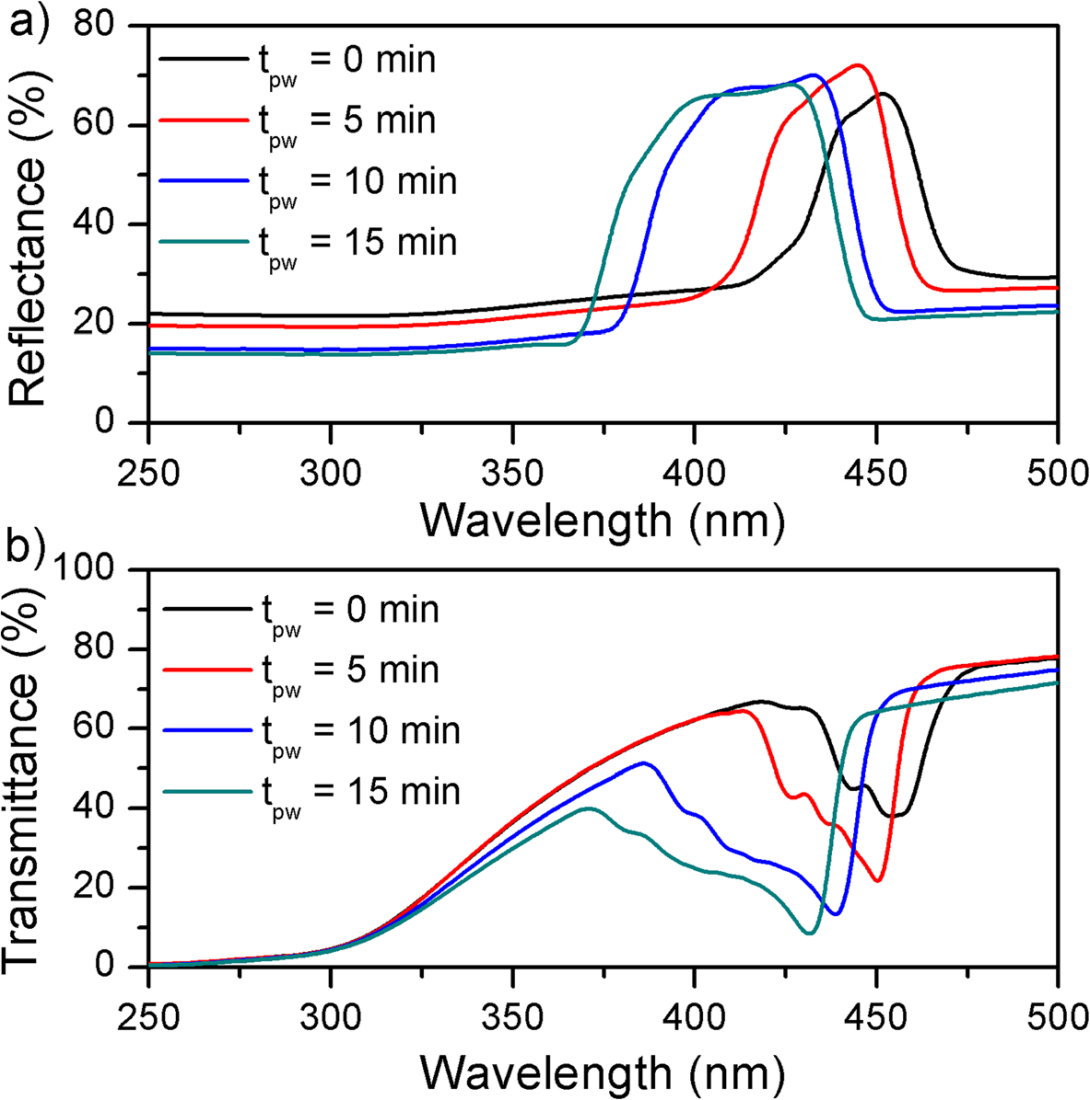
**Reflectance and transmittance characterization of the NAA rugate filters. (a)** Reflectance and **(b)** transmittance spectra of NAA rugate filters anodized for 300 cycles, with an apodized sinusoidal current profile with a period time of *T* = 200 s.

In order to assess the effect of porosity on the NAA rugate filter, we fabricated four sets of samples with a period of *T* = 200 s and applied a pore-widening post-treatment for 0, 5, 10, and 15 min. The remaining Al was selectively dissolved to ensure that the reflection observed was only due to the rugate structure. Figure 4a shows the resulting reflectance spectra. The spectra displayed a well-defined band without sidelobes as we expected from the apodization of the current profile. We observed that the pore-widening treatment resulted in a blueshift of the reflection band as well as a lower reflection below and above the band. This is the result of the partial dissolution of the alumina, which decreases the overall refractive index of the rugate filter. A more interesting fact is how the band widened after the pore-widening treatment. This broadening is related to the refractive index contrast of the rugate filter (Δ*n*). The higher the Δ*n*, the wider the band. This is in good agreement with our previous reported results for NAA obtained with periodic anodization voltages [7,14]. Analysis of the transmittance measurements (Figure 4b) showed how the pore-widening post-treatment led to less steep edges in the stop band, possibly due to scattering and absorption of the alumina.

### Real-time sensing

**Figure 5:**
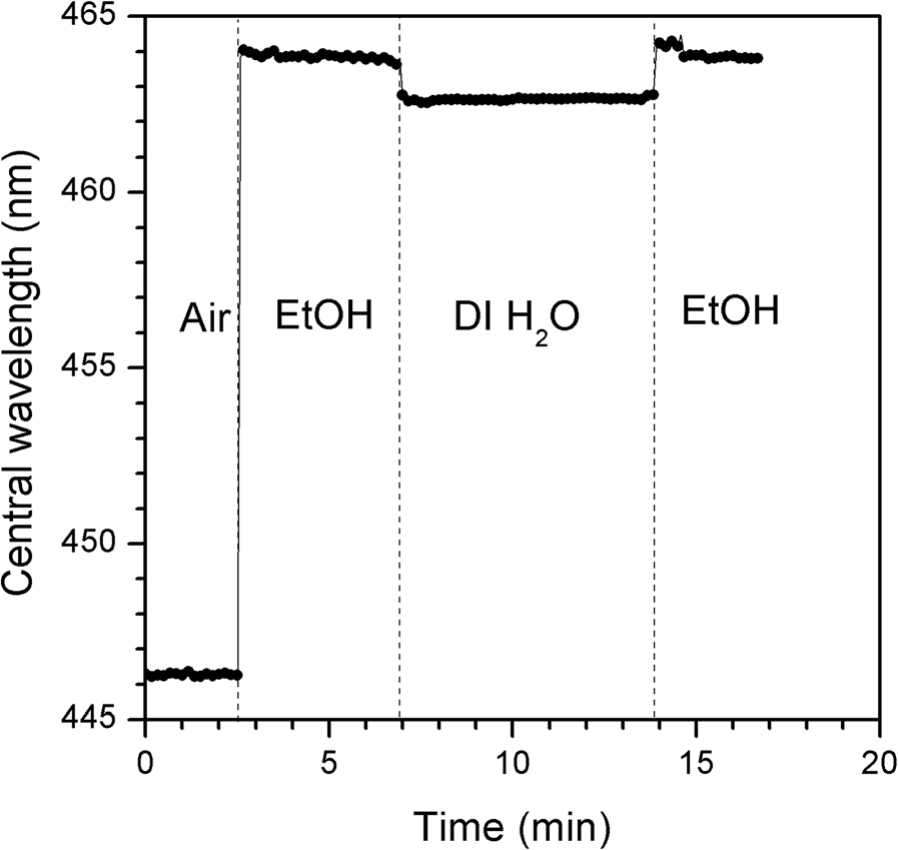
**Sensing results.** Real-time measurement of a NAA rugate filter in a flow-cell where EtOH, deionized water, and EtOH were serially flushed in to the chamber.

As a proof of the possible application of this structure, we performed a sensing experiment in a flow cell and monitored the position of the reflectance band in real-time for a sample fabricated with a period time of *T* = 200 s, a total of 300 cycles, and a pore-widening post-treatment of *t*_pw_ = 5 min (Figure 5). After acquiring a reference of the sample in air, we flowed EtOH at a rate of 1 mL min^−1^. Then, we flowed deionized water and, finally, EtOH again in order to prove the repeatability of the measurement. The results presented in Figure 5 show a highly stable signal with no significant drift within the time range and a very low noise of about 0.04 nm. The NAA rugate filter was able to distinguish between two liquids with a similar refractive index (*n*_water_ = 1.333, *n*_EtOH_ = 1.362) with a sensitivity of 48.8 nm/refractive index unit (RIU). Moreover, when EtOH was reintroduced into the chamber, the position of the reflection band returned to the same value of the first EtOH infiltration, indicating the high reproducibility of the results.

## Conclusions

NAA rugate filters were fabricated using a current control method based on a sinusoidal current profile with a maximum amplitude of just 1.45 mA cm^−2^. Thanks to this small current peak-to-peak value, the voltage was contained within 40 ± 5 V. The position of the band can be accurately tuned by varying the period time of the current profile. This process allows the fabrication of highly reflective bands with just 50 periods. Moreover, for as-produced rugate filters, the reflectance bands were narrow (less than 30 nm) which is an important feature for the development of highly sensitive chemical and biochemical sensors based on the monitorization of the position of the reflectance band. As a proof of concept, we performed a sensing experiment in a flow cell in order to determine the sensing possibilities of the structure and found out that changes in refractive index of 0.031 can be readily monitored with high sensitivity (48.8 nm/RIU) and low noise level (<0.04 nm).

## Abbreviations

DBR: distributed Bragg reflector; NAA: nanoporous anodic alumina; PBG: photonic band gap; pSi: porous silicon; RIU: refractive index unit.

## Competing interests

The authors declare that they have no competing interests.

## Authors’ contributions

GM, LFM, and JFB designed the experiment and analyzed and discussed the results. GM fabricated the NAA rugate filters, performed the optical characterization, and redacted the manuscript. JFB, JP, and LFM revised the manuscript. All authors approved the final manuscript.
